# Development of Hallux Valgus Classification Using Digital Foot Images with Machine Learning

**DOI:** 10.3390/life13051146

**Published:** 2023-05-09

**Authors:** Mitsumasa Hida, Shinji Eto, Chikamune Wada, Kodai Kitagawa, Masakazu Imaoka, Misa Nakamura, Ryota Imai, Takanari Kubo, Takao Inoue, Keiko Sakai, Junya Orui, Fumie Tazaki, Masatoshi Takeda, Ayuna Hasegawa, Kota Yamasaka, Hidetoshi Nakao

**Affiliations:** 1 Department of Rehabilitation, Osaka Kawasaki Rehabilitation University, Mizuma 158, Kaizuka 597-0104, Japan; 2 Graduate School of Rehabilitation, Osaka Kawasaki Rehabilitation University, Mizuma 158, Kaizuka 597-0104, Japan; 3 Graduate School of Life Science and Systems Engineering, Kyushu Institute of Technology, Hibikino 2-4, Wakamatsu-ku, Kitakyushu 808-0135, Japan; eto.shinji786@mail.kyutech.jp (S.E.);; 4 Department of Industrial Systems Engineering, National Institute of Technology, Hachinohe College, 16-1 Uwanotai, Tamonoki, Hachinohe 039-1192, Japan; 5 Department of Rehabilitation, Takata-Kamitani Hospital, Kamiyamaguchi 4-26-14, Yamaguchi, Nishinomiya 651-1421, Japan; 6 Department of Physical Therapy, Josai International University, 1 Gumyo, Togane 283-8555, Japan

**Keywords:** hallux valgus, machine learning, image classification, VGG16, accuracy, preprocessing

## Abstract

Hallux valgus, a frequently seen foot deformity, requires early detection to prevent it from becoming more severe. It is a medical economic problem, so a means of quickly distinguishing it would be helpful. We designed and investigated the accuracy of an early version of a tool for screening hallux valgus using machine learning. The tool would ascertain whether patients had hallux valgus by analyzing pictures of their feet. In this study, 507 images of feet were used for machine learning. Image preprocessing was conducted using the comparatively simple pattern A (rescaling, angle adjustment, and trimming) and slightly more complicated pattern B (same, plus vertical flip, binary formatting, and edge emphasis). This study used the VGG16 convolutional neural network. Pattern B machine learning was more accurate than pattern A. In our early model, Pattern A achieved 0.62 for accuracy, 0.56 for precision, 0.94 for recall, and 0.71 for F1 score. As for Pattern B, the scores were 0.79, 0.77, 0.96, and 0.86, respectively. Machine learning was sufficiently accurate to distinguish foot images between feet with hallux valgus and normal feet. With further refinement, this tool could be used for the easy screening of hallux valgus.

## 1. Introduction

Hallux valgus (HV) is a common foot deformity characterized by static subluxation of the first metatarsophalangeal joint with lateral deviation of the hallux and medial deviation of the first metatarsal [1]. High prevalence is also a characteristic of HV; 23% of adults aged 18–65 years were reportedly affected, with incidence increasing with age and being higher in women than in men [2]. Increased severity of HV is associated with decreased health-related quality of life, physical function, balance function, and risk of falls [3,4,5,6,7]. HV progresses over time, so early intervention should be sought to prevent the onset of severe HV. A large HV angle is said to be a risk factor for the progression of HV deformity [8].

HV is generally diagnosed based on the first-second intermetatarsal angle and HV angle, both of which are usually determined by radiography [9]. Radiography is typically performed in the hospital, and patients with HV who present to the hospital likely already have advanced deformity, pain, and other symptoms. In addition, radiologic examination requires some degree of radiation exposure. Early intervention for HV is important to maintain physical function and to prevent worsening leading to disability. General practitioners in Australia, for example, encounter an estimated 60,000 bunions each year, so there is a problematically high medical economic burden [10]. A simple method to measure HV in community-dwelling adults at home without the need for a hospital visit could therefore be useful. Home blood pressure monitoring, for example, has helped to reduce cardiovascular disease events. Similarly, home screening tools for HV may help to prevent HV becoming severe [11]. 

The Manchester scale (MS), a relative nonmetric measurement of severity of HV, has potential use within a tool for community-dwelling adults to screen HV severity by themselves [12]. In MS, HV is visually classified into four levels (no deformity, mild deformity, moderate deformity, severe deformity). The MS has a statistically significant correlation with the American Orthopaedic Foot and Ankle Society score [13]. It can be measured in a clinical setting, and it has been reported to correlate moderately well with radiography [14]. However, for the early detection and prevention of severe disease, a tool than can more easily assess HV is desired. This could be achieved by image classification using machine learning. Image classification using machine learning is currently being actively studied in the medical field, and it can be a supplementary tool for diagnosis based on radiography and computed tomography images [15]. 

Image recognition using images taken with digital cameras has been successfully used to screen for sarcopenia [16]. However, a similar tool to screen for HV has not yet been reported. We therefore developed a non-invasive tool that can screen HV according to foot images using a machine learning framework, and we examined its accuracy. The tool classifies foot images into ‘normal’ (no deformity, mild deformity) and HV (moderate deformity, severe deformity) based on the MS. This classification is based on the suggestion that HV moderate deformity and severe deformity groups are considered to have reduced foot function, general foot health, and social competence [17].

## 2. Materials and Methods

### 2.1. Study Materials

**Figure 1:**
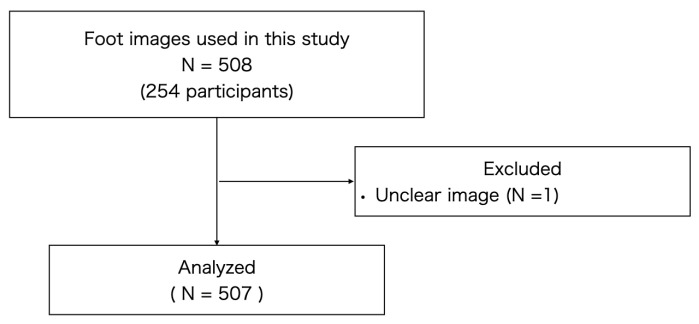
Flowchart illustrating the selection of study materials.

We used 508-foot images obtained from 254 participants of a local government-supported check-up for health in Kaizuka City, Osaka, Japan. One of the images was excluded from the analysis because it was unclear (Figure 1). All participants lived at home and were independent in terms of their activities in daily living. 

### 2.2. Severity of Hallux Valgus

The severity of HV was determined by the MS using images of the foot. Patients stood in a full-weight-bearing position and the degree of HV was recorded as either no deformity, mild deformity, moderate deformity, or severe deformity. This was visually determined by two physiotherapists, each of whom have more than 20 years of experience. There were determined to be 243 images of no deformity and 61 images of mild deformity, which made up the normal group. There were 122 images of moderate deformity and 81 images of and severe deformity, which made up the HV group (Table 1).

### 2.3. Foot Imaging

**Figure 2:**
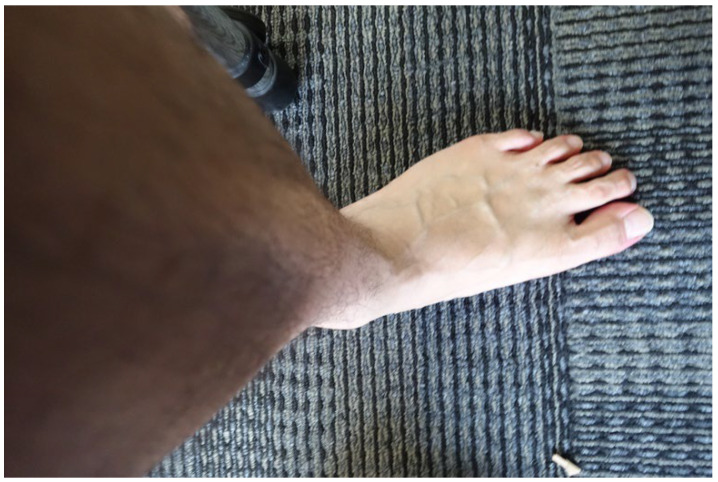
Example of foot image.

Images of feet were taken at three health and welfare centers by trained research staff. To ensure uniform imaging conditions, participants stepped onto a movable box and were asked to place their weight evenly on both feet in a standing position. The mobile box was 90 cm high, and its top was covered with a black sheet. A digital camera (RX-0, SONY, Tokyo, Japan) was placed at the height of the participant’s tibial tuberosity and images were taken of the feet from above (Figure 2). The digital camera features 20.3 megapixels, a focal length of 4.3 (W)—172.0 mm, a resolution of 5184 × 2912 pixels, and one pixel in a captured image is 0.005 square centimeters.

**Figure 3:**
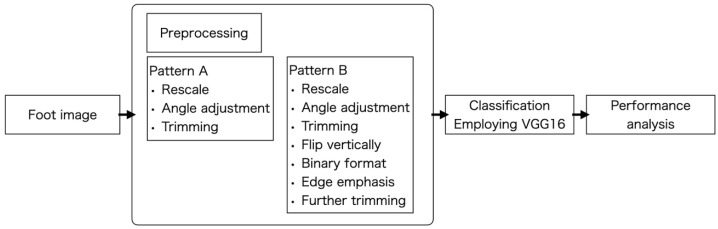
Comprehensive framework for HV classification.

Images were preprocessed as follows (Figure 3). Preprocessing was performed in two patterns to verify whether the degree of accuracy varies with the degree of preprocessing.

**Figure 4:**
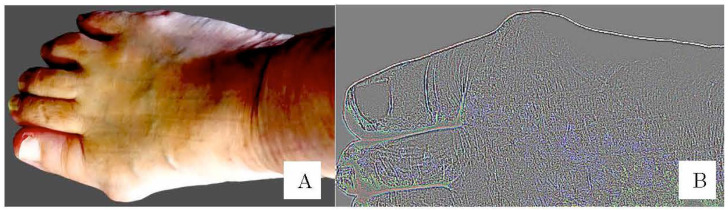
Image preprocessing. (**A**) Pattern A: The images had the background removed and were horizontally corrected. The image was further cropped to include only the metatarsus from the toes. (**B**) Pattern B: The image was vertically inverted, and the edges were enhanced.

Pattern A (Figure 4A)

(1) The image was converted to a size width of 640 pixels and the background was digitally removed. The foot image was adjusted to horizontal orientation. The image was cropped so that only the area from the toes to the midfoot area was included.

Pattern B (Figure 4B)

Pattern B was preprocessed in the same way as pattern A, with the following additions:

(2) The right foot image was vertically inverted to match the orientation of the left and right feet.
(3) The image was converted to grayscale, the edges were enhanced, and the image was cropped so that only the midfoot area from the tip of the hallux was included.
(4) The normal group (n = 304) and the HV group (n = 203) have different numbers of images, which could negatively affect the accuracy. Data augmentation was therefore performed on the images in the HV group. This involved changing the contrast and saturation of 101 randomly selected images in the HV group. Color jittering, such as varying of saturation and contrast, is common in data augmentation used in machine learning research [18].

Comparison of the difference in accuracy between patterns A and B is necessary because it may aid in decision-making in future applications. It may be necessary to restrict the imaging conditions in which the subject themself judges the HV. Hypothetically, in the future this could be a lay person rather than a specialist. Restriction of the imaging conditions may also affect the complexity when programming. Image preprocessing was performed manually using the picture tool on a personal computer (iMAC, Apple M1, 16 GB memory) and a photo editor application, PhotoScape X (MOOII Tech, Seoul, Republic of Korea).

### 2.4. Classification by Machine Learning

Machine learning was performed with the following hardware and software. Central processing unit: Apple M1, Memory: 16 GB, operating system: macOS 13.2.1, Framework: TensorFlow, Keras, Python 2.11.0. This study used VGG16 for Convolutional Neural Network. VGG16 consists of 13 convolutional layers and three convolutional layers, totaling 16 layers, and is used in image classification research [19,20,21,22]. The number of training epochs was 20, the batch size was 16, and the image size of the input layer was downsized to 224 × 224 pixels. The activation and loss functions were Adam and cross-entropy, respectively.

### 2.5. Statistical Analysis

Foot images were randomly assigned to groups of training data (80%) and validation data (20%). Confusion matrix was used to determine how well the model performs against validation data. The performance of the image classification model for HV identification was evaluated in terms of accuracy, precision, recall, and F1 score. Accuracy is the most common model evaluation metric in machine learning and is easy to interpret and understand. Precision is a measure of the ability of a machine learning model to predict accurately. The F1 score measures the sensitivity of precision and sensitivity. It was introduced to solve the conflict between precision and recall. Recall is a percentage value that suggests how well a positive class was predicted; it focuses on positivity for the correct answer [23]. The performance of the confusion matrix and image classification models was analyzed and compared for both A and B patterns of image preprocessing.

## 3. Results

Table 2 shows the confusion matrix of the HV image classification model used in this study. 

Using pattern A, of the HV images, 72 images were correctly distinguished as HV and 157 images were incorrectly distinguished as Normal. Of the Normal images, 13 images were incorrectly distinguished as HV and 206 images were correctly distinguished as Normal. 

Using pattern B, of the HV images, 102 images were correctly distinguished as HV and 128 images were incorrectly distinguished as Normal. Of the Normal images, 28 images were incorrectly distinguished as HV and 423 images were correctly distinguished as Normal.

Pattern A of the model used in this study achieved scores of 0.62 for accuracy, 0.56 for precision, 0.94 for recall, and 0.71 for F1 score. Pattern B of the model used in this study achieved 0.79 for accuracy, 0.77 for precision, 0.96 for recall, and 0.86 for F1 score (Table 3).

## 4. Discussion

Few studies of machine learning with photography have been conducted in medical research. A small number of studies have been performed on its use in classification of acromegaly, skin cancer, and the severity of caries [24,25,26]. This study is the first to demonstrate that a machine learning framework can discriminate foot deformity in humans. 

We applied an existing machine learning framework to verify whether it is possible to identify HV from digital images. The results suggest that HV can be identified by deep learning with appropriate image preprocessing. The pattern B machine learning model for identifying HV demonstrated accuracy of 0.79, precision of 0.77, recall of 0.96, and an F1 score of 0.86. HV has not been identified using machine learning in previous studies, making the comparison of accuracy of the models used in this study difficult. However, there are existing studies in which there was image analysis using machine learning in orthopedics [27,28]. The accuracy of these models to detect arthritis and trauma from magnetic resonance imaging (MRI) and radiography was 75–92.8%. Based on these previous orthopedics studies, the accuracy of the model constructed in this study is thought to be acceptable.

Compared with pattern B, the pattern A machine learning model for identifying HV was less accurate, perhaps owing to the comparative simplicity of the image preprocessing. To identify HV using machine learning, we suggest that vertical flipping, edge emphasis, and trimming are necessary. However, the confusion matrix of pattern B demonstrated high accuracy in distinguishing normal foot images as normal, but low accuracy in distinguishing HV. Further improved image preprocessing may achieve better accuracy. Machine learning was previously implemented for the diagnosis of hip osteoarthritis, achieving accuracy of 92.8% [29]. That study used magnified images focusing upon osteophytes. The diagnosis of knee osteoarthritis using a deep Siamese convolution neural network had average accuracy of 66.7% and kappa coefficient of 0.83 [30]. Elsewhere, feature value extraction with machine learning has been used in the detection of cancer and COVID-19 using MRI images and radiography [31,32]. The purpose of this study was to develop a screening tool for HV, so we did not use radiography or MRI images. However, we suggest that improved accuracy in identifying MS-based HV might be obtained with additional preprocessing, feature value extraction, and greater emphasis on the first metatarsophalangeal joint.

The main limitation of this study is that it was not possible to use all four gradings of the MS. This limitation may be solved by collecting more images in the future, and the current accuracy needs to be improved. Improved image preprocessing is needed to achieve greater accuracy. This machine learning does not account for the complexity of the deformity and associated conditions, such as pes planus. Nonetheless, this study showed that, to some extent, machine learning models could identify HV from images captured by digital cameras without the use of radiography. Applications of such machine learning models could be used within tools that allow community-dwelling adults to easily identify whether they have HV without the need for consultation with a medical professional. This could contribute to alleviation of the health–economic problem of HV.

## 5. Conclusions

Machine learning tools may be able to detect whether or not a foot image is HV. Accuracy will be increased by improving image preprocessing. After further development, a similar tool could be used to screen the severity of hallux valgus.

## Figures and Tables

**Table 1:** Classification of foot images used in this study based on the MS.

Category	Number	Classification
No deformity	243	Normal
Mild deformity	61	
Moderate deformity	122	Hallux valgus
Severe deformity	81	

MS: Manchester Scale.

**Table 2:** Confusion matrix of machine learning models to identify HV.

Pattern A		Prediction	
		HV	Normal
Result	HV	72	157
	Normal	13	206
**Pattern B**		**Prediction**	
		**HV**	**Normal**
Result	HV	102	128
	Normal	28	423

HV: Hallux valgus.

**Table 3:** Accuracy of Machine Learning Models with Different Preprocessing.

	Accuracy	Precision	Recall	F1 Score
Pattern A	0.62	0.56	0.94	0.71
Pattern B	0.79	0.77	0.96	0.86

## Data Availability

Not applicable.
